# Modeling of High-Density Compaction of Pharmaceutical Tablets Using Multi-Contact Discrete Element Method

**DOI:** 10.3390/pharmaceutics13122194

**Published:** 2021-12-18

**Authors:** Kostas Giannis, Carsten Schilde, Jan Henrik Finke, Arno Kwade

**Affiliations:** 1 Center of Pharmaceutical Engineering (PVZ), Technische Universität Braunschweig, Franz-Liszt-Str. 35A, 38106 Braunschweig, Germany; c.schilde@tu-braunschweig.de (C.S.); jan.finke@tu-braunschweig.de (J.H.F.); a.kwade@tu-braunschweig.de (A.K.); 2 Institute for Particle Technology (iPAT), Technische Universität Braunschweig, Volkmaroder Str. 5, 38104 Braunschweig, Germany

**Keywords:** compaction, multi-contact DEM, plastic deformation, MCC, tableting

## Abstract

The purpose of this work is to simulate the powder compaction of pharmaceutical materials at the microscopic scale in order to better understand the interplay of mechanical forces between particles, and to predict their compression profiles by controlling the microstructure. For this task, the new framework of multi-contact discrete element method (MC-DEM) was applied. In contrast to the conventional discrete element method (DEM), MC-DEM interactions between multiple contacts on the same particle are now explicitly taken into account. A new adhesive elastic-plastic multi-contact model invoking neighboring contact interaction was introduced and implemented. The uniaxial compaction of two microcrystalline cellulose grades (Avicel^®^ PH 200 (FMC BioPolymer, Philadelphia, PA, USA) and Pharmacel^®^ 102 (DFE Pharma, Nörten-Hardenberg, Germany) subjected to high confining conditions was studied. The objectives of these simulations were: (1) to investigate the micromechanical behavior; (2) to predict the macroscopic behavior; and (3) to develop a methodology for the calibration of the model parameters needed for the MC-DEM simulations. A two-stage calibration strategy was followed: first, the model parameters were directly measured at the micro-scale (particle level) and second, a meso-scale calibration was established between MC-DEM parameters and compression profiles of the pharmaceutical powders. The new MC-DEM framework could capture the main compressibility characteristics of pharmaceutical materials and could successfully provide predictions on compression profiles at high relative densities.

## 1. Introduction

The ability to predict the bulk behavior of granular materials is of great importance for many industrial applications (i.e., tableting, metal forming) when deformation has to be handled in a controlled manner. Pharmaceutical powders are a branch of granular materials and while undergoing high loaded compression under confined conditions, allow the formation of compact granules, especially tablets, a process known as compaction or tableting. Pharmaceutical powder compaction is a crucial production process for pharmaceutical manufacturing due to the prevalence of tablets as solid dosage forms. Understanding the compaction behavior is of practical importance to improve the efficiency of product development and the manufacturing performance [1,2].

Powder compaction is frequently modeled using either continuous or discrete numerical techniques, or a combination of both. On the one hand, the finite element method (FEM) is a continuum approach that allows for the representation of deformation at a larger scale when combined with a suitable constitutive law, such as the Drucker–Prager cap (DPC) [3] or modified DPC [4], but does not reveal the physics of the system at the particle level. For this reason, one way to model the mechanical response of discrete elements at a particle level is the application of the multiple particle finite element method (MPFEM) [5,6,7] where each individual particle is being meshed with finite elements. Another way is to combine FEM and discrete element method (DEM), referred as meshed discrete element method (MDEM) [8,9], an approach that uses the contact detection algorithm from DEM and applies it in the FEM context. The capability to consider particles as deformable bodies is the key benefit of these approaches. The critical problem is their computational complexity, which prevents them from being used in large-scale industrial operations.

On the contrary, granular materials are mathematically described as a collection of particles by using the soft discrete element method (DEM) [10], and the bulk behavior of the granular materials is determined based on the interactions between pairs of particles. Newton’s second law of motion is used to define the trajectories of each particle, and particle deformation is proportional to the overlap between particles that are in contact. When an overlap is detected, an appropriate contact model is called, which associates the overlap with the force experienced at the contact point.

The DEM is the only currently available technique that can provide insight at the particle level and has been used in a wide variety of applications, such as ceramics [11], pharmaceutical, and food industries [12,13]. Given that the DEM is an excellent tool for studying these applications, modeling of confined powder compression with DEM remains a challenge. Based on Hertz’s theory, particles are considered to be rigid bodies to which deformation occurs locally and concentrated at the contact points. However, the basic assumptions of Hertz’s theory (classical DEM) is limited on capturing small deformations [14] and is rendered invalid for large strains, which typically occur under high relative densities and high loads. As a result, Hertz’s contact theory must be extended or modified to account for the fact that a larger (flattening) contact area results in a higher contact stiffness. To address this issue, many existing classical DEM contact models have added contact stiffness depending on plastic deformation and flattening in the contact areas. Ganrer et al. [15] proposed another adhesive elastic-plastic contact model to simulate powder compaction. The authors calibrated the contact stiffness between-particles (p-p) and particles-walls (p-w) using simulations of mono-sized particles, to reach high relative densities, at the macro-scale, and to predict the compression profiles. In a similar way, Y. Gao et al. [16]. applied the Luding’s elastioplastic [17] contact model to model powder compaction. Coarse-grained particles were used in this method and contact stiffness between particles (and p-w) was calibrated for one material under uni-axial compaction and the calibrated values were then used to predict the compression profiles of mixtures of additional materials.

**Figure 1:**
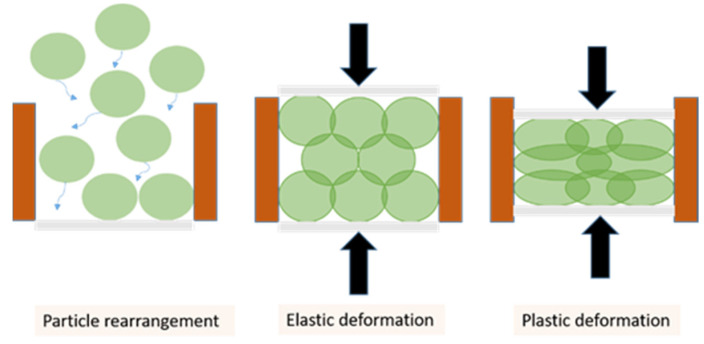
The three phases of a packing of simultaneously deforming particles.

In fact, many of the existing classical DEM contact models regard each contact of the same particle as independent of neighboring contacts, which is reasonable for loose powders but unrealistic for high relative densities. Three important characteristics of the mechanical behavior of elastic-plastic contacts are induced by a packing of simultaneously deforming particles undergoing uni-axial compaction (Figure 1): (a) the die filling and rearranging of the particles in the first phase; (b) the deformation is initially elastic, at this phase the contact areas between the particles are small with each contact independent of its neighbors; (c) the material yields entering the plastic zone and, because the pores are now almost closed the spatial confinement creates high contact pressures that allows for an additional degree of resistance that becomes more significant with time. Fischmeister and Arzt [18] refer to this as “geometrical hardening”. At this last phase, the compact is pressed to (nearly) full density [19], and mechanically behaves like a porous solid. The development of contact pressure originates from changes in the crystal lattices or intermolecular interactions. It has been suggested that contact dependency arises at relative densities of 0.7 and higher [14,20,21]. In practice, a relative density higher than 0.8 is required to produce commercial pharmaceutical tablets with adequate mechanical strength. As a result, multiple-contact DEM contact models are necessary.

In this regard, researchers have made efforts on the formulation of the multi-contact discrete element method (MC-DEM) as an attempt to implicitly introduce particles’ deformability. One way is to enhance Hertz’s elastic contact theory and rewrite its classical equations such that not only local particle deformation but also the global deformation is taken into account. The global deformation is defined as the result of multiple contacts imposed on a single particle by neighboring particles. Brodu et al. [22] suggested a technique wherein the strain field acting on a single particle is coupled with the classical Hertz’s equation to account for the global deformation. Brodu et al. [22], validated their novel model by predicting the compression profiles of a packing of hydrogel balls compressed at low stresses. As an alternative, the stress field might be used in the equations. As a result, a multi-contact model that takes contact dependency into account was proposed by Frenning [23]. The particle global deformation was related to the isotropic stress tensor in this case. Giannis et al. [24] introduced a stress-based multi-contact model that takes anisotropic particle deformation into account and was validated for relevant materials with in the elastic regime. Attempts have also been made to explicitly introduce deformable particles in the framework of the DEM in order to address the basic assumptions of Hertz’s theory. A sophisticated model was presented by Rojek et al. [25] proposed the approach of the so-called deformable discrete element method (DDEM). This method is conceptually similar to the method provided by Brodu et al. [22]. The main difference is that particle deformability is introduced explicitly with the DDEM. The per particle stress tensor generates the isotropic particle deformation. As a result, the new deformable shape induces the formation of new contact points (not accessible with classical DEM). The main issues of this method are that (a) only isotropic particle deformation is considered, and (b) the high computing cost limits its use to 2D cases [25] or 3-D [26] cases with a small number of particles.

This work is divided into two parts: (a) using an extension of Giannis et al. [24] multi-contact model, to consider plastic deformation of pharmaceutical particles, and (b) to compare the results of the experiments with model calculations based on the MC-DEM framework.

The article’s outline is as follows: Section 2.1 contains the basic equations of the classical formulation of the DEM; multi-contact modeling is being discussed in Section 2.2 of this document; the materials used in this study are given in Section 3 of this article; Section 4 presents the calibration strategy and numerical results; and the last section, concludes with some final thoughts.

## 2. DEM’s Theoretical Background

Particle deformations are reproduced in soft-particle DEM by overlaps between interacting particles. When an overlap is detected, a contact law is used to compute the contact forces (force–displacement) between two particles. The underlying assumption is that particle contacts are independent of one another, and therefore contact forces are resolved locally. Newton’s equations of motion are used in this method to determine the connection between particle motion and forces acting on each particle. The equations of a particle’s translational and rotational motion are:

(1) $$m_{i}{\ddot{a}}_{i}=\underset{j}{\sum }\left(F_{nij}+F_{tij}\right)+m_{i}g\;\;\;and\;\;I_{i}{\dot{\omega }}_{i}=\tau_{ij}$$

where $m_{i}$, ${\ddot{a}}_{i}$, $I_{i}$ and ${\dot{\omega }}_{i}$ are the mass, acceleration, moment of inertia and angular velocity for particle i, respectively; $F_{nij}$, $F_{tij}$, $\tau_{ij}$ are the normal force, tangential force, and torque acting on particles *i* and *j* at contact points, respectively; g is the acceleration due to gravity.

Different contact models can be used to express force–displacement laws at contact points. This study does not go into great depth on the various contact models and their related equations. Rojek [27] and Thakur [28] summarize the many contact models that are used in discrete particle simulations. In their works, O’Sullivan [29] and Thornton [30] go into deep details on contact models.

### 2.1. Classical Hertz–Mindlin Contact Model

**Figure 2:**
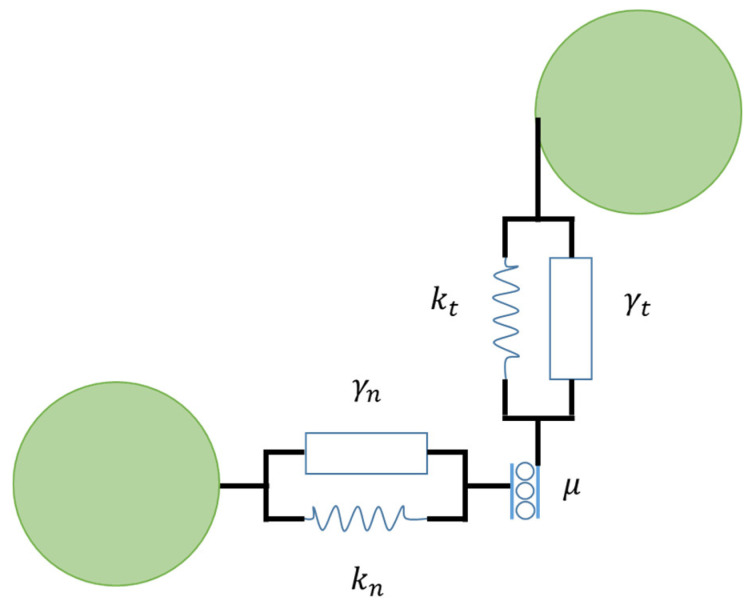
Contact force model illustrating particle interaction with normal and tangential forces.

The linear spring-dashpot model [31], in which the spring stiffness is considered to be constant, is the simplest contact configuration. To enhance the linear contact model, the Hertz theory [32] (classical) is used to calculate the force-displacement relation for contacting particles (e.g., nonlinear spring-dashpot model). In this case, the normal stiffness varies depending on the degree of overlap. The Hertz–Mindlin [33,34,35,36] contact model is another contact model for representing the force-displacement relation. This nonlinear model combines accuracy and simplicity of the Hertz theory in the normal direction with the Mindlin model in the tangential direction. This model includes a contact force as well as a viscous contact damping force at contact points. In the normal (*n*) and tangential (*t*) axes, these forces were calculated using elastic springs and dashpots (Figure 2). The normal repulsive contact force is:

(2) $$F_{n}=k_{n}\delta_{n}^{3/2}+\gamma_{n}\;{\dot{\delta }}_{n}$$

where $k_{n}=\frac{4}{3}E^{*}\sqrt{R^{*}}$ is the normal stiffness coefficient, with $R^{*}=\frac{R_{i}R_{j}}{R_{i}+R_{j}}$ the effective radius and $E^{*}=\frac{1-\nu_{i}^{2}}{E_{i}}+\frac{1-\nu_{j}^{2}}{E_{j}}$ is the effective Young’s modulus. In this expression, ν and G represent the particles Poisson’s ratio and shear modulus, respectively. The normal overlap is $\delta_{n}$, ${\dot{\delta }}_{n}$ is the relative velocity in normal direction of interacting particles and $\gamma_{n}$ the viscoelastic damping constant for normal contact viscosity. The tangential force is [24]:

(3) $$F_{t}=k_{t}\delta_{t}^{3/2}+\gamma_{t}\;{\dot{\delta }}_{t}$$

where $k_{t}=8G^{*}\sqrt{R^{*}}$ is the tangential stiffness coefficient and $G^{*}=\frac{2-\nu_{i}}{G_{i}}+\frac{2-\nu_{j}}{G_{j}}$ the effective shear modulus. The tangential overlap is $\delta_{t}$, ${\dot{\delta }}_{t}$ is the relative velocity in tangential direction of interacting particles and $\gamma_{t}$ the viscoelastic damping constant for tangential contact viscosity.

The tangential overlap, $\delta_{t}$, between particles obtained by integrating surface relative tangential velocities during elastic deformation of the contact is given as [37,38]:

(4) $$\delta_{t}=\int_{t}^{t+\Delta t}v^{t}dt\to \;\delta_{t}\;\approx \;v^{t}dt$$

where $v^{t}$ is the velocity component tangential to the contact surface and Δt is the time-step.

The tangential and normal forces are connected by Coulomb’s law, $F_{t}\leq \mu F_{n}$, in the event of sliding, there is dynamic friction. $F_{t}=\mu F_{n}$. The dynamic and static friction coefficients are assumed to be equal in this case., $\mu =\mu_{d}=\mu_{s}$. In order to allow for a restoring force, a static situation requires the use of an elastic spring, i.e., a non-zero remaining tangential force in static equilibrium due to activated Coulomb friction. By applying a torque to the contacting surfaces, rolling friction can be controlled. The rolling friction constant directional torque (CDT), $\tau_{ij}$, used for this study, is given by [39]:

(5) $$\tau_{ij}=-\frac{\omega_{rel}}{\left|\omega_{rel}\right|}\mu_{r}R_{r}F_{n}$$

The particles in these models are assumed to be spherical and do not deform during simulation. In a strict sense, it is assumed that particles are undergoing some kind of pseudo deformation, and this model is known as the truncated Hertz–Mindlin model [40]. Moreover, these models include binary interactions between two particles, which implies that during contacts, particles are in touch through a single point.

### 2.2. Multi-Contact Adhesive Elastic-Plastic Model

The linear or non-linear elasticity theory, on the other hand, is only applicable to small deformations. However, when it comes to powder compaction, plasticity prevails (flattening in contact areas), necessitating the modeling of elastic-plastic spheres in contact. As a result, Hertz theory must be extended to cases in which particles are deformed plastically. Persson and Frenning [40], for instance, presented an extension of classical Hertz theory to account for elastic-plastic contacts. In this example, a limiting contact pressure was added, whereas adhesion was not added, such that plastic deformation begins after the contact region’s maximum pressure is achieved.

We propose a novel adhesive elastic-plastic multi-contact model that combines concepts from the Luding [17] and Edinburgh [28] adhesive elastic-plastic (hysteretic) contact models and the multi-contact model proposed by Giannis et al. [24], the pseudo-code of the algorithm utilized is briefly described in the Appendix A. For the first time, this model is being used in this study to investigate the behavior of elastic-plastic medicinal materials. When two particles collide with one another elastic and plastic deformation (linear and non-linear force-displacement curve) occur. A nonlinear contact model is proposed that takes into consideration both elastic-plastic contact deformation and adhesion. The adhesive plastic force is:

(6) $$F_{n}=\{\;\begin{array}{l} F_{0}+k_{1}\delta_{n}^{3/2}\;\;\;\;\;\;\;\;\;\;if\;\;k_{2}\left(\delta_{n}^{3/2}-\delta_{0}^{3/2}\right)\geq k_{1}\delta_{n}^{3/2}\\ F_{0}+k_{2}^{*}\left(\delta_{n}^{3/2}-\delta_{0}^{3/2}\right)\;\;if\;\;k_{1}\delta_{n}^{3/2}>k_{2}^{*}\left(\delta_{n}^{3/2}-\delta_{0}^{3/2}\right)>-k_{c}\delta_{n}^{3/2}\\ F_{0}-k_{c}\delta_{n}^{3/2}\;\;\;\;\;\;\;\;\;\;if\;-k_{c}\delta_{n}^{3/2}\geq k_{2}^{*}\left(\delta_{n}^{3/2}-\delta_{0}^{3/2}\right) \end{array}$$

**Figure 3:**
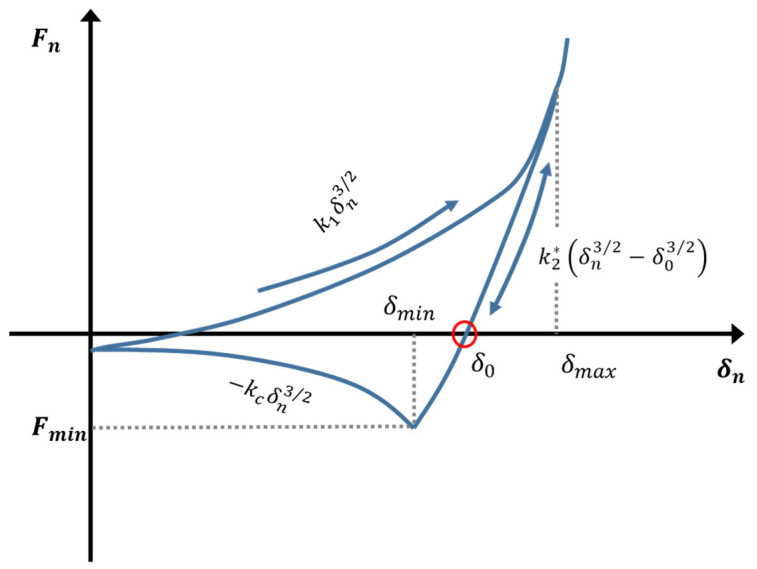
A non-linear hysteretic, adhesive force-displacement (δ) relation in normal direction. The slope $k_{2}^{*}$ of the unloading and reloading branch interpolates between $k_{1}$ and a maximum stiffness $k_{2}$.

Figure 3 illustrates the force-displacement curve. The term “displacement” refers to the overlap of particles. The loading, unloading, re-loading, and adhesive branches *e* is defined by the loading branch stiffness $k_{1}$, the loading-unloading branch stiffness $k_{2}$, the adhesion branch stiffness $k_{c}$, the plastic overlap (deformation) $\delta_{0}$ and the constant pull-off force $F_{0}$. During initial loading the contact model follows the virgin loading path $k_{1}$, until the maximum overlap is reached at $\delta_{max}$. The maximum overlap $\delta_{max}$ is a contact-specific history-dependent parameter that is updated and saved. During unloading the contact will alter from virgin loading $k_{1}$ to unloading/reloading $k_{2}$, which depends on $\delta_{max}$. At $\delta_{max}$, the force is decreasing from its value to zero at overlap $\delta_{0}$, which resembles the plastic contact deformation (remaining overlap). The plastic overlap is defined as:

(7) $$\delta_{ο}={\left(1-\frac{k_{1}}{k_{2}}\right)}^{\frac{2}{3}}\delta_{max}$$

for cases where the limit is met, with $k_{1}=k_{2}$ results in $\delta_{ο}=0\;$(no remaining overlap) yields to a special case of non-linear elasticity. Hence, the non-linear elastic Hertz–Mindlin contact model is included as a special case. On the other hand, $k_{2}\to \infty$ captures a perfectly plastic contact. Unloading below $\delta_{ο}$ results in attractive adhesion forces until the minimum force is equal:

(8) $$\;F_{min}=-k_{c}\delta_{min}^{3/2}$$

And the overlap $\delta_{min}$ is:

(9) $$\delta_{min}={\left(\frac{k_{2}-k_{1}}{k_{2}-k_{c}}\right)}^{\frac{2}{3}}\delta_{max}$$

Attractive forces emerge as the unloading process continues.

(10) $$F_{n}=-k_{c}\delta_{n}^{3/2}$$

In order to account for the fact that a larger contact surface leads to a higher contact stiffness, the coefficient $k_{2}$ is made dependent on the maximum overlap $\delta_{max}$ (history dependent parameter):

(11) $$k_{2}^{*}\left(\delta_{max}\right)=\{\begin{array}{l} k_{2}\;\;\;\;\;\;\;\;\;\;\;\;\;\;\;\;if\;\delta_{max}\geq \delta_{max}^{*}\\ k_{1}+\left(k_{2}-k_{1}\right)\frac{\delta_{max}^{*}}{\delta_{max}}\;\;\;\;if\;\delta_{max}<\delta_{max}^{*}\;\; \end{array}$$

The behavior of the unloading slope described by Equation (11) is similar to that assumed by Luding [10,17], with the exception that nonlinear behavior is addressed here. Likewise, the limit of plastic flow overlap is given:

(12) $$\;\delta_{max}^{*}=\frac{k_{2}}{k_{2}-k_{1}}\varphi_{f}\frac{2R_{i}R_{j}}{R_{i}+R_{j}}$$

where $\varphi_{f}$ is the dimensionless plasticity depth, defined in relation to the reduced radius. The original contact model proposed by Luding can be characterized as a piecewise linear hysteretic model [17]. For the virgin loading, the contact normal stiffness $k_{1}$ and normal overlap $\delta_{n}$ is used to calculate the force. While stiffness $k_{1}$ is not a physical parameter according to Luding’s contact model, in this work it is depending on the Young’s modulus. The new stiffness is $k_{n}=\frac{4}{3}E^{*}\sqrt{R^{*}}\;$ is identical to the one of Hertz theory (Section 2.1) and similar to the one of Edinburgh contact model. Furthermore, in contrast with Luding’s and in line with the Edinburgh [28] contact model, a non-linear force-displacement relation is proposed $F_{n}=k_{1}\delta_{n}^{3/2}$. By contrast, the unloading stiffness $k_{2}$ is load dependent as in Luding’s model. Additionally, the new contact model was supplied with a non-linear adhesion $F_{n}=-k_{c}\delta_{n}^{3/2}$.

Moreover, to address the fundamental assumption of the classical DEM, which treats each contact locally as a binary pair interaction, Giannis et al. [24] presented a nonlocal model which takes into account the mutual influence between contacts. While this model has been verified for cases in the elastic regime, in this study we will extend its applicability to capture plasticity.

**Figure 4:**
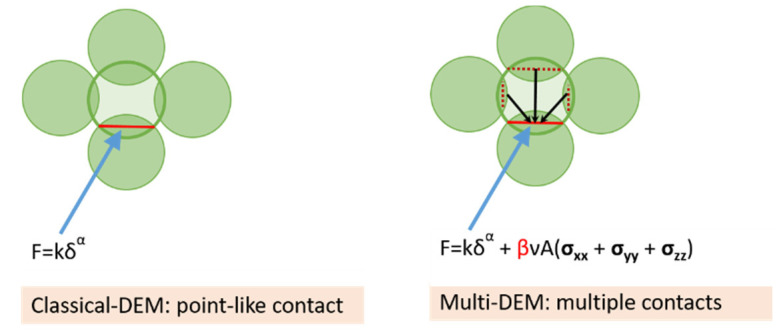
A multi-contact modification of the classical DEM.

The main idea of the on how to account for multi contact effect is shown in Figure 4. Multiple contacts acting on a particle have been taken into account by using the trace of the average stress tensor coupled with the Poisson’s ratio (***v***), the contact area (***A***) between interacting particles, and a material-dependent prefactor (***β***). More information may be found here [24]. The new multi-contact law formulation yields to this equation:

(13) $$F_{n}=k_{n}\delta_{n}^{3/2}+\left(\beta \nu A_{ij}\right)P_{ij}$$

The first term in Equation (13) was defined before in Section 2.1 of this article (Equation (2)). The second term carries the information from neighboring particles operating on the particle. In this expression, β is a dimensionless empirical prefactor that allows for particle geometry changes to be taken into consideration indirectly, ν is the Poisson’s ratio, and $A_{ij}$ is the contact are between the interacting particles. The isotropic component of the stress is the pressure $P_{ij}=\frac{1}{3}\left(tr\left(\sigma_{i}\right)+tr\left(\sigma_{j}\right)\right)$, with $tr\left(\sigma \right)=\left(\sigma_{xx}+\sigma_{yy}+\sigma_{zz}\right)$ and $\sigma_{i},\;\sigma_{j}$ the stress tensors of particle i and j, respectively. By combining Equations (6) and (13), the multi-contact model can be extended from linear to plastic deformation, yielding a new equation:

(14) $$F_{n}=\{\begin{array}{l} F_{0}+k_{1}\delta_{n}^{3/2}\;\;\;\;\;\;\;\;+\left(\beta \nu A_{ij}\right)P_{ij}\;\;if\;\;k_{2}\left(\delta_{n}^{3/2}-\delta_{0}^{3/2}\right)\geq k_{1}\delta_{n}^{3/2}\\ F_{0}+k_{2}^{*}\left(\delta_{n}^{3/2}-\delta_{0}^{3/2}\right)+\left(\beta \nu A_{ij}\right)P_{ij}\;\;if\;\;k_{1}\delta_{n}^{3/2}>k_{2}^{*}\left(\delta_{n}^{3/2}-\delta_{0}^{3/2}\right)>-k_{c}\delta_{n}^{3/2}\\ F_{0}-k_{c}\delta_{n}^{3/2}\;\;\;\;\;\;\;+\left(\beta \nu A_{ij}\right)P_{ij}\;\;if\;-k_{c}\delta_{n}^{3/2}\geq k_{2}^{*}\left(\delta_{n}^{3/2}-\delta_{0}^{3/2}\right) \end{array}$$

In the next sections, this new equation (Equation (14)) will be investigated and verified for the modeling cases of uni-axial compaction of pharmaceutical materials.

## 3. Materials and Methods

### 3.1. Materials

Two microcrystalline cellulose grades Avicel^®^ PH 200 (FMC BioPolymer, Philadelphia, PA, USA) and Pharmacel^®^ 102 (DFE Pharma, Nörten-Hardenberg, Germany) were studied in depth. Henceforth, the powders shall be referred to by the abbreviations MCC-A and MCC-P. A certain proportion of the MCC particles, particularly the larger particle sizes, are rounded agglomerates. Taking this into account, and due to the simplicity of this approach in simulation, spherical shapes are used in DEM simulations. The powder characteristics particle size distribution (PSD) and true density, are given in Table 1 and are available in the literature [41].

### 3.2. Experimental Methods

Compaction experiments were performed applying a Styl’One evolution compaction simulator (CS; Medel’Pharm, Beynost, France). This equipment can accurately control the compaction process and allows for in-depth investigation of powder properties and to extract force/displacement profiles. In-die data were evaluated by applying the software ANALIS (Medel’Pharm, Beynost, France). Generic profile was applied for compaction to reach compression stresses of approx. 30, and 180 MPa.

### 3.3. Numerical Methods

In a wide range of applications, including this study, the discrete element method (DEM) is used to model and analyze granular materials. However, predictions can only be correct if the input parameter values are carefully chosen. There are a number of input parameters that need to be tuned depending on the contact model used; this procedure is known as calibration. An in-depth review into calibration is provided by [42,43,44,45]. If Luding’s [17] original contact model is to be used, a total of 19 input parameters must be predefined or calibrated. A comprehensive experimental determination of these parameters would be highly time-consuming and labor-intensive to accomplish. Fortunately, not all parameters have the same effect on the simulation output. As a consequence, only the parameters that are most significant to the validity of the simulation results are considered. Material parameters for the single particles and used in this study were obtained from the study of Cabiscol et al. [41]. The calibration technique consists of bibliographical sources, experiments, and their replication by DEM simulations. DEM simulations of nano-indentation experiments were used to determine the fitting parameter that expresses the identical experimental results, in terms of force and displacement; therefore the Young’s modulus (E) of a single particle may be determined. The ring shear tester and the Jenike wall test were used for a direct determination of the sliding friction between particles (μ_s(pp)_) and between particles and walls (μ_s(pw)_). Later, the tumbling drum test was used to determine the final frictional and rotational DEM related parameters μ_s(pw)_, μ_r(pp)_, and μ_r(pw)_. This test functioned as a calibration test for the rolling friction as well as a second and final iteration for μ_s(pw)_, starting from the values obtained from the ring shear tester and the Jenike wall test. The complete calibration method is described in depth in the Cabiscol et al. study [41]. The DEM input parameters for the material properties are summarized in Table 1. The calibration of the input parameters of the new multi-contact model will be discussed in Section 4.1.1.

## 4. Results and Discussion

### 4.1. Determination of a Representative Volume Element (RVE)

Full-scale DEM simulations need a significant amount of computational power; to bypass this limitation, a representative volume element is used. The literature has several definitions of the representative volume element (RVE), the most notable of which are discussed in [46,47,48,49]. Although there is no single and exact definition of the RVE, the basic concept is that the RVE should be large enough to retain the microstructural information while being small enough in relation to the macroscopic structural dimensions to eliminate fluctuations. In this study, the RVE concept was used to speed up the simulations. The method used to determine an RVE was similar to that described by Wiącek et al. [50]. The basic ideas are as follows: (a) set an initial packing contained in a small domain (the smallest possible); (b) gradually expand the domain’s dimensions while maintaining the same particle size distribution (PSD) and packing density; (c) carry out numerical simulations to obtain the force-displacement curve; and (d) analyze the results to determine if they are converging.

**Figure 5:**
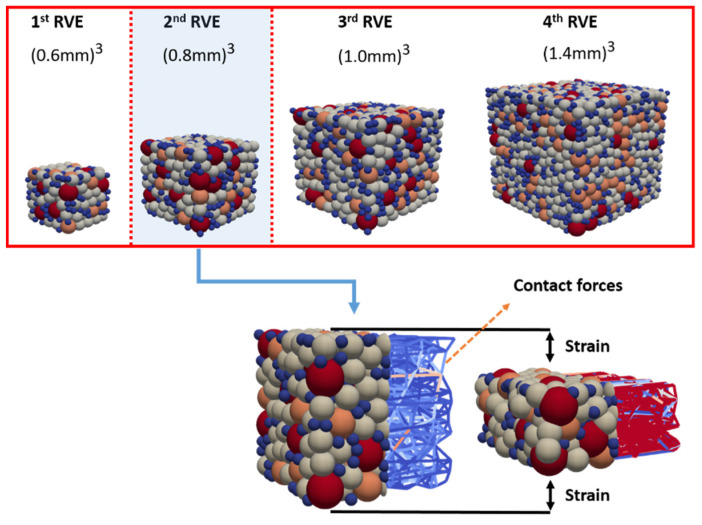
Determination of a representative volume element (RVE).

**Figure 6:**
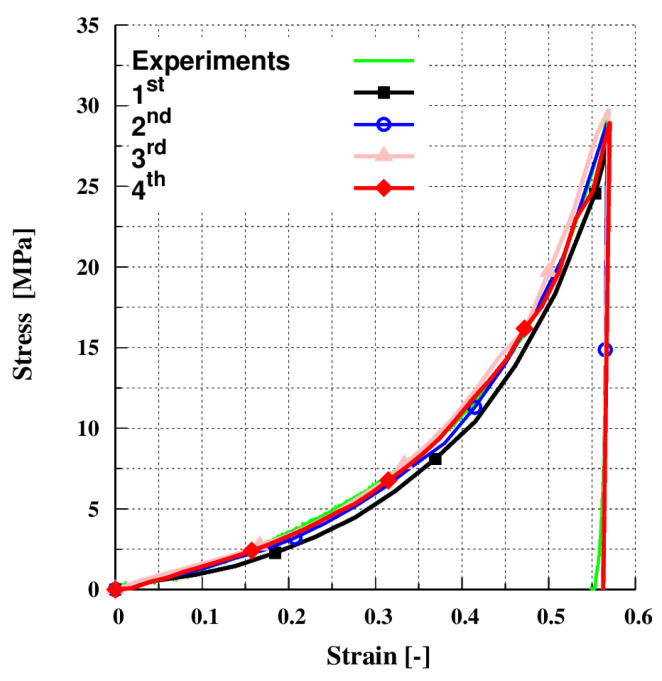
Converging analysis of the suggested RVEs.

The simulations presented here involve a series of uni-axial compaction tests in cubes of the following ranging sizes: (0.6 mm)^3^ (1stRVE), (0.8 mm)^3^ (2nd RVE), (1.0 mm)^3^ (3nd RVE), and (1.4 mm)^3^ (4th RVE) (see Figure 5). To eliminate the wall boundary effect, periodic boundaries were used along the X and Y axes. The cubes contain a top and a bottom plate. A series of uniaxial compression simulations were performed using our new multi-contact DEM model. After calibration, as will be discussed later in Section 4.1.1, and based on the data shown in Figure 6 we can confirm the existence of an RVE since the results are converging. However, due to the small size of the sample, the results of the first RVE underestimated its macroscopic stress–strain response. There is excellent agreement between the results of the following RVEs, as well as with the experimental data. It is therefore decided to use the second RVE for practical reasons (less computational time) as follows.

#### 4.1.1. Calibration Method for the Input Parameters of the Multi-Contact Model

**Figure 7:**
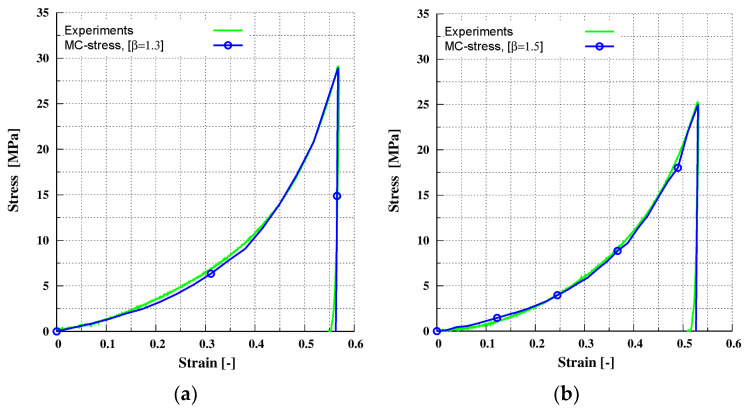
Calibration for the: (**a**) MCC-A material under uni-axial compaction at maximum target stress of 29 MPa; (**b**) MCC-P material under uni-axial compaction at maximum target stress of 25 MPa.

In this section, the calibration for the new multi-contact model is shown. In this case, and as discussed in Section 4.1, the system under consideration is a cube with dimensions (0.8 mm)^3^ (2nd RVE in Figure 5) along x-y-z directions. The system under consideration contains 698 particles for the MCC-A material and 1193 particles for the MCC-P material with a particle size distribution for both materials given in Section 3.1 and Table 1. The particles are initially randomly positioned in a cubic system with periodic boundary constraints in order to minimize wall effects. After initial deposition, the particles are allowed to grow. Growth is terminated as soon as the desired packing density of approximately 59% is reached and is in line with experimental results reported in the literature [41,51]. The sample was then compressed uni-axially along the z-axis to a maximum target strain of 57% for MCC-A and 53% for MCC-P, then it was decompressed. A strain-driven simulation was used to achieve the maximum desired stress of 29 MPa for the MCC-A material and 25 MPa for the MCC-P material. The calibrated material parameters presented in Table 2 (Section 3.2) were used here. However, the input parameters for the multi-contact model were obtained using an iterative process to determine the optimum parameters that better fits the experimental results. A parameter optimization method was used based on a series of simulations, similar to the one presented by Gao et al. [16]. Given the experimental macroscopic stress and strain response the ***R***^2^ value between the experimental and simulated data was calculated from:

(15) $$R^{2}=1-\frac{\sum^{\;}{\left(y-\hat{y}\right)}^{2}}{\sum^{\;}{\left(y-\bar{y}\right)}^{2}}$$

where y indicates the stress response of the experimental data, $\hat{y}$ indicates the stress response of the simulated data and $\bar{y}$ indicates the mean of the stress response of the experimental data. The value of ***R***^2^ was used to evaluate the accuracy with which the simulated input parameters fit the experimental data; a successful fit was attained when ***R***^2^ was close to 1. Therefore, when ***R***^2^ exceeded 0.95, the iterations needed for calibration were terminated. Figure 7 shows that experimental and simulated results are in excellent agreement, indicating that the calibration was successful. Table 3 summarizes the input parameters that were calibrated. For the dimensionless plasticity depth $\varphi_{f}$, a high and constant value was selected (low contact stiffness) to achieve a high contact stiffness; when necessary, the prefactor β was tuned accordingly.

#### 4.1.2. Verification for Uni-Axial Compaction for MCC-A

**Figure 8:**
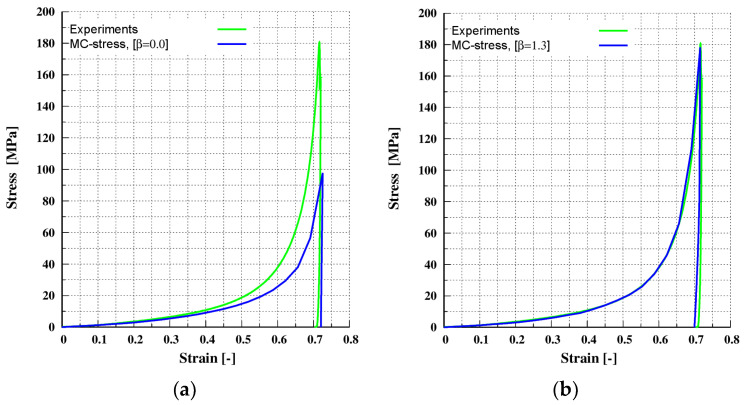
Verification for the MCC-A material under uni-axial compaction at maximum target stress of 180 MPa: (**a**) without the multi-contact effect (prefactor β = 0.0); (**b**) with the multi-contact effect (prefactor β = 1.3).

In this section, the simulation results for compaction of the MCC-A material are shown. The system is identical to the one presented in Section 4.1.1. The sample was compressed uni-axially along the z-direction to a maximum target strain of 71%, and then decompressed. The target stress for this case is 180 MPa. The calibrated material input parameters given in Section 3.2 (Table 2) and, for the multi-contact DEM model given in Section 4.1.1 (Table 3), were used. When the calibrated prefactor β = 1.3 (Section 4.1.1 (Table 3)) was used, an excellent agreement between experimental and simulated results was achieved, as shown in Figure 8b. A value of prefactor β = 0.0 indicates that the multi-contact effect is not included and when β = 0.0 conventional DEM underestimates the macroscopic stress–strain response, as shown in Figure 8a. It is also clear from comparing Figure 8a,b the multi-contact effect predominates for strains higher than 0.2.

#### 4.1.3. Verification for Uni-Axial Compaction for MCC-P

**Figure 9:**
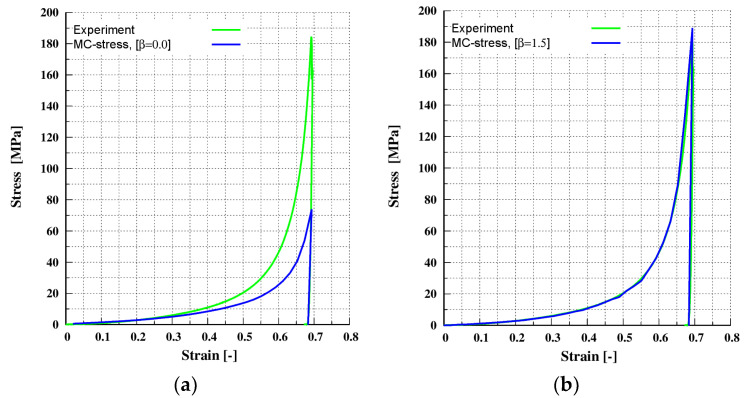
Verification for the MCC-P material under uni-axial compaction at maximum target stress of 185 MPa: (**a**) without the multi-contact effect (prefactor β = 0.0); (**b**) with the multi-contact effect (prefactor β = 1.3).

In this section, the simulation results for compaction of the MCC-P material are shown. The system is identical to the one presented in Section 4.1.1 for the MCC-P material. The sample was compressed uni-axially along the z-direction to a maximum target strain of 69%, and then decompressed. The target stress for this case is 185 MPa. The calibrated material input parameters given in Section 3.2 (Table 2) and, for the multi-contact DEM model given in Section 4.1.1 (Table 3), were used. When the calibrated prefactor β = 1.5 (Section 4.1.1 (Table 3)) was used, an excellent agreement between experimental and simulated results was achieved, as shown in Figure 9b. As expected with β = 0.0 conventional DEM underestimates, the macroscopic stress–strain response is shown in Figure 9a. It is also clear from comparing Figure 9a,b that the multi-contact effect predominates for strains higher than 0.2, around the same point as that seen for a the MCC-A material in Section 4.1.2.

## 5. Conclusions

In this study, by employing our new elastic-plastic multi-contact DEM model, the compaction profiles (stress–strain) of microcrystalline cellulose grades Avicel^®^ PH 200 (FMC BioPolymer, Philadelphia, PA, USA) and Pharmacel^®^ 102 (DFE Pharma, Nörten-Hardenberg, Germany) were successfully predicted. It was also shown that the multi-contact effect predominates for strains higher than 0.2. A calibration strategy to calibrate the input parameters, prefactor β, for the multi-contact model was presented here. The prefactor β was calibrated at low relative densities (low macroscopic stress) and subsequently used for high relative densities (high macroscopic stress). The new multi-contact model requires a separate calibration for each material as prefactor β is a material-dependent parameter. However, more research is needed to determine if this is also true for a mixture of other relevant materials.

In terms of the input parameters for the multi-contact model prefactor β, the unloading stiffness $\kappa_{2}$ and the cohesion stiffness $\kappa_{c}$ were the only parameters that were calibrated. The loading stiffness $\kappa_{1}$ was related to the Young’s modulus of the material. The material input parameters were calibrated separately. The concept of a representative volume element (RVE) was used to speed up simulations. In comparison to alternative approaches that use coarse-grained particles, the RVE was preferred because the particle size distribution (PSD) can be maintained while using the RVE.

In future work, we will aim to calibrate the prefactor β as an intrinsic material parameter. The aim is to conduct uni-axial compaction simulation in a series of relevant materials, then create a comprehensive database, and finally, with the assistance of artificial intelligence (e.g., neural network), generalize the results. Furthermore, the results presented here are based on the assumption of perfect spherical particles; in a future attempt, the real shape of the particles should be addressed.

## Figures and Tables

**Table 1:** Powder characteristics: PSD and densities [41].

Material	*x*_10_ (*Q*_3_) (μm)	*x*_50_ (*Q*_3_) (μm)	*x*_90_ (*Q*_3_) (μm)	Span (-)	True Density (kg m^−3^)
MCC-A	82.9	224.6	379.3	1.32	1541.1
MCC-P	28.3	86.5	173.8	1.68	1533.7

**Table 2:** The input parameters for single particles and walls [41].

Property	Symbol	Units	MCC-A	MCC-P
Young’s modulus—particle (p)	E	Nm^−2^	2.58 × 10^8^	1.34 × 10^9^
Young’s modulus—wall (w)	E	Nm^−2^	7.62 × 10^10^	7.62 × 10^10^
Poisson’s ratio—particle	ν	-	0.30	0.30
Poisson’s ratio—wall	ν	-	0.31	0.31
Coefficient of restitution particle	COR(p-p)	-	0.352	0.346
Coefficient of restitutio—wall	COR(p-w)	-	0.352	0.346
Coefficient of sliding fric—(p-p)	μ_s(pp)_	-	0.561	0.548
Coefficient of sliding f—(p-w)	μ_s(pw)_	-	0.707	0.715
Coefficient of rollin—(p-p)	μ_r(pp)_	-	0.3	0.3
Coefficient of rol—(p-w)	μ_r(pp)_	-	0.01	0.01
Density	ρ	kg/m^3^	1541.1	1533.7

**Table 3:** Multi-contact model input parameters.

Property	Symbol	Units	MCC-A	MCC-P
Unloading stiffness	k_2_/k_1_	-	120	120
Adhesion stiffness ratio	K_c_/k_1_	-	0.5	0.5
Dimensionless plasticity depth	φ_f_	-	0.99	0.99
Prefactor of the MC-dem	β	-	1.3	1.5

**Figure A1:**
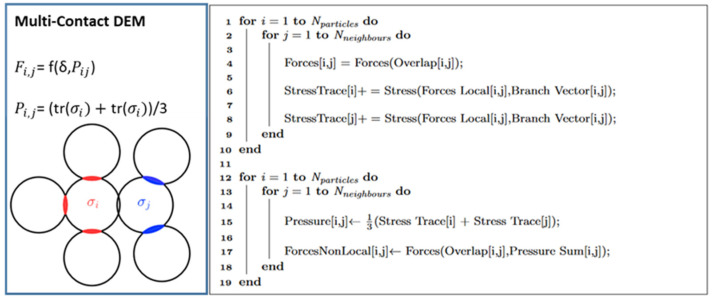
Pseudo-code used in to obtain the non-local contact forces acting on a particle.

## Data Availability

Not applicable.

## References

[1] Martin N.L., Schomberg A.K., Finke J.H., Abraham T.G., Kwade A., Herrmann C. (2021). Process Modeling and Simulation of Tableting—An Agent-Based Simulation Methodology for Direct Compression. Pharmaceutics.

[2] Wünsch I., Finke J.H., John E., Juhnke M., Kwade A. (2019). Mathematical Approach to Consider Solid Compressibility in the Compression of Pharmaceutical Powders. Pharmaceutics.

[3] Diarra H., Mazel V., Busignies V., Tchoreloff P. (2017). Comparative study between Drucker-Prager/Cap and modified Cam-Clay models for the numerical simulation of die compaction of pharmaceutical powders. Powder Technol..

[4] Ohsaki S., Kushida K., Matsuda Y., Nakamura H., Watano S. (2020). Numerical study for tableting process in consideration of compression speed. Int. J. Pharm..

[5] Gethin D.T., Lewis R.W., Ransing R.S. (2003). A discrete deformable element approach for the compaction of powder systems, Modelling Simul. Mater. Sci. Eng..

[6] Procopio A.T., Zavaliangos A. (2005). Simulation of multi-axial compaction of granular media from loose to high relative densities. J. Mech. Phys. Solids.

[7] Demirtas A., Klinzing G.R. (2021). Understanding die compaction of hollow spheres using the multi-particle finite element method (MPFEM). Powder Technol..

[8] Stránský J., Jirásek M. Open Source FEM–DEM Coupling. Proceedings of the 18th International Conference Engineering Mechanics.

[9] Frenning G. (2008). An efficient finite/discrete element procedure for simulating compression of 3D particle assemblies. Comput. Methods Appl. Mech. Eng..

[10] Luding S. (2008). Introduction to discrete element methods. Eur. J. Environ. Civ. Eng..

[11] Iacobellis V., Radhi A., Behdinan K. (2019). Discrete element model for ZrB_2_-SiC ceramic composite sintering. Compos. Struct..

[12] Horabik J., Wiącek J., Parafiniuk P., Stasiak M., Bańda M., Kobyłka R., Molenda M. (2020). Discrete Element Method Modelling of the Diametral Compression of Starch Agglomerates. Materials.

[13] Raji A.O., Favier J.F. (2004). Model for the deformation in agricultural and food particulate materials under bulk compressive loading using discrete element method. I: Theory, model development and validation. J. Food Eng..

[14] Harthong B., Jérier J.-F., Dorémus P., Imbault D., Donzé F.-V. (2009). Modeling of high-density compaction of granular materials by the Discrete Element Method. Int. J. Solids Struct..

[15] Garner S., Strong J., Zavaliangos A. (2018). Study of the die compaction of powders to high relative densities using the discrete element method. Powder Technol..

[16] Gao Y., de Simone G., Koorapaty M. (2021). Calibration and verification of DEM parameters for the quantitative simulation of pharmaceutical powder compression process. Powder Technol..

[17] Luding S. (2008). Cohesive, frictional powders: Contact models for tension. Granul. Matter.

[18] Fischmeister H.F., Arzt E. (1983). Densification of Powders by Particle Deformation. Powder Metall..

[19] Olsson E., Larsson P.-L. (2013). A numerical analysis of cold powder compaction based on micromechanical experiments. Powder Technol..

[20] Mesarovic S.D., Fleck N.A. (2000). Frictionless indentation of dissimilar elastic–plastic spheres. Int. J. Solids Struct..

[21] Jonsson H., Gråsjö J., Frenning G. (2017). Mechanical behaviour of ideal elastic-plastic particles subjected to different triaxial loading conditions. Powder Technol..

[22] Brodu N., Dijksman J.A., Behringer R.P. (2015). Multiple-contact discrete-element model for simulating dense granular media. Phys. Rev. E.

[23] Frenning G. (2013). Towards a mechanistic model for the interaction between plastically deforming particles under confined conditions: A numerical and analytical analysis. Mater. Lett..

[24] Giannis K., Schilde C., Finke J.H., Kwade A., Celigueta M.A., Taghizadeh K., Luding S. (2021). Stress based multi-contact model for discrete-element simulations. Granul. Matter.

[25] Rojek J., Zubelewicz A., Madan N., Nosewicz S. (2018). The discrete element method with deformable particles. Int. J. Numer. Methods Eng..

[26] Rojek J., Nosewicz S., Thoeni K. (2021). 3D formulation of the deformable discrete element method. Int. J. Numer. Methods Eng..

[27] Rojek J., Popp A., Wriggers P. (2018). Contact Modeling in the Discrete Element Method. Contact Modeling for Solids and Particles.

[28] Thakur S.C. (2014). Mesoscopic Discrete Element Modelling of Cohesive Powders for Bulk Handling Applications.

[29] O’Sullivan C. (2011). Particulate Discrete Element Modelling.

[30] Thornton C. (2015). Granular Dynamics, Contact Mechanics and Particle System Simulations: A DEM Study.

[31] Cundall P.A., Strack O.D.L. (1979). A discrete numerical model for granular assemblies. Géotechnique.

[32] Hertz H. (2009). Ueber die Berührung Fester Elastischer Körper. J. Reine Angew. Math..

[33] Mindlin R.D. (1949). Compliance of Elastic Bodies in Contact. J. Appl. Mech..

[34] Mindlin R.D., Deresiewicz H. (1953). Elastic Spheres in Contact under Varying Oblique Forces. J. Appl. Mech..

[35] Di Renzo A., Di Maio F.P. (2004). Comparison of contact-force models for the simulation of collisions in DEM-based granular flow codes. Chem. Eng. Sci..

[36] Di Renzo A., Di Maio F.P. (2005). An improved integral non-linear model for the contact of particles in distinct element simulations. Chem. Eng. Sci..

[37] Shäfer J., Dippel S., Wolf D.E. (1996). Force Schemes in Simulations of Granular Materials. J. Phys. I.

[38] Silbert L.E., Ertaş D., Grest G.S., Halsey T.C., Levine D. (2002). Geometry of frictionless and frictional sphere packings. Phys. Rev. E.

[39] Ai J., Chen J.-F., Rotter J.M., Ooi J.Y. (2011). Assessment of rolling resistance models in discrete element simulations. Powder Technol..

[40] Persson A.-S., Frenning G. (2012). An experimental evaluation of the accuracy to simulate granule bed compression using the discrete element method. Powder Technol..

[41] Cabiscol R., Finke J.H., Kwade A. (2019). Assessment of particle rearrangement and anisotropy in high-load tableting with a DEM-based elasto-plastic cohesive model. Granul. Matter.

[42] Cabiscol R., Finke J.H., Kwade A. (2018). Calibration and interpretation of DEM parameters for simulations of cylindrical tablets with multi-sphere approach. Powder Technol..

[43] Coetzee C.J. (2017). Review: Calibration of the discrete element method. Powder Technol..

[44] Simons T.A., Weiler R., Strege S., Bensmann S., Schilling M., Kwade A. (2015). A Ring Shear Tester as Calibration Experiment for DEM Simulations in Agitated Mixers—A Sensitivity Study. Procedia Eng..

[45] Paulick M., Morgeneyer M., Kwade A. (2015). A new method for the determination of particle contact stiffness. Granul. Matter.

[46] Gitman I.M., Askes H., Sluys L.J. (2007). Representative volume: Existence and size determination. Eng. Fract. Mech..

[47] Rojek J., Karlis G.F., Malinowski L.J., Beer G. (2013). Setting up virgin stress conditions in discrete element models. Comput. Geotech..

[48] Drosopoulos G.A., Giannis K., Stavroulaki M.E., Stavroulakis G.E. (2018). Metamodeling-Assisted Numerical Homogenization for Masonry and Cracked Structures. J. Eng. Mech..

[49] Montero F., Medina F. Determination of the RVE Size of Quasi-Brittle Materials Using the Discrete Element Method. Proceedings of the II International Conference on Particle-Based Methods-Fundamentals and Applications PARTICLES 2011.

[50] Wiącek J., Molenda M., Ooi J.Y., Favier J. (2012). Experimental and numerical determination of representative elementary volume for granular plant materials. Granul. Matter.

[51] Nordström J., Alderborn G., Frenning G. (2018). Compressibility and tablet forming ability of bimodal granule mixtures: Experiments and DEM simulations. Int. J. Pharm..

